# Testing the role of ancient and contemporary landscapes on structuring genetic variation in a specialist grasshopper

**DOI:** 10.1002/ece3.2810

**Published:** 2017-03-30

**Authors:** Víctor Noguerales, Pedro J. Cordero, Joaquín Ortego

**Affiliations:** ^1^ Grupo de Investigación de la Biodiversidad Genética y CulturalInstituto de Investigación en Recursos Cinegéticos ‐ IREC (CSIC, UCLM, JCCM)Ciudad RealSpain; ^2^ Department of Integrative EcologyEstación Biológica de Doñana (EBD‐CSIC)SevilleSpain

**Keywords:** Bayesian inference, climate niche modeling, genetic diversity, genetic structure, isolation by resistance, topographic complexity

## Abstract

Understanding the processes underlying spatial patterns of genetic diversity and structure of natural populations is a central topic in evolutionary biogeography. In this study, we combine data on ancient and contemporary landscape composition to get a comprehensive view of the factors shaping genetic variation across the populations of the scrub‐legume grasshopper (*Chorthippus binotatus binotatus*) from the biogeographically complex region of southeast Iberia. First, we examined geographical patterns of genetic structure and employed an approximate Bayesian computation (ABC) approach to compare different plausible scenarios of population divergence. Second, we used a landscape genetic framework to test for the effects of (1) Late Miocene paleogeography, (2) Pleistocene climate fluctuations, and (3) contemporary topographic complexity on the spatial patterns of population genetic differentiation. Genetic structure and ABC analyses supported the presence of three genetic clusters and a sequential west‐to‐east splitting model that predated the last glacial maximum (LGM,* c*. 21 Kya). Landscape genetic analyses revealed that population genetic differentiation was primarily shaped by contemporary topographic complexity, but was not explained by any paleogeographic scenario or resistance distances based on climate suitability in the present or during the LGM. Overall, this study emphasizes the need of integrating information on ancient and contemporary landscape composition to get a comprehensive view of their relative importance to explain spatial patterns of genetic variation in organisms inhabiting regions with complex biogeographical histories.

## 1 Introduction

Understanding the mechanisms that shape spatial patterns of genetic diversity and structure is a central topic in evolutionary biogeography (Habel et al., 2015; Peterman, Connette, Semlitsch, & Eggert, 2014; Yannic et al., 2014). Present‐day landscape configuration and the geographical distribution of suitable habitats, jointly with species‐specific ecological characteristics, define contemporary interpopulation dispersal and realized gene flow (Castillo, Epps, Davis, & Cushman, 2014; Edwards, Keogh, & Knowles, 2012). However, past climate changes and ancient geological events have also greatly altered the spatial configuration of corridors and barriers to gene flow (He, Edwards, & Knowles, 2013; Pepper, Doughty, Arculus, & Keogh, 2008). Such temporal shifts in landscape structure and dispersal routes have often left genetic signatures in contemporary populations that are useful to track back in time their past demographic trajectories (He et al., 2013; Lanier, Massatti, He, Olson, & Knowles, 2015). Thus, the study of how present‐day and past landscape composition have impacted gene flow is necessary to get a comprehensive view of the processes underlying spatial patterns of genetic diversity and structure of natural populations, which can ultimately help to predict their responses to ongoing or future environmental changes (Fordham, Brook, Moritz, & Nogués‐Bravo, 2014; Yannic et al., 2014).

Quaternary climatic fluctuations, characterized by cold glacial stages alternated with warm interglacial periods, have strongly influenced the demography of many organisms during the past 2 million years (2–0.04 Mya) (Hewitt, 2000). During glacial periods, the distribution ranges of most species from temperate zones contracted and their populations persisted in refugia located at lower elevations or latitudes (Homburg et al., 2013; Qu et al., 2014). Conversely, the populations from cool‐adapted species expanded during glacial periods and shrank during interglacials (Canestrelli & Nascetti, 2008). Under any scenario, populations from regions subjected to major climate changes experience fluctuating demographic dynamics that, ultimately, are expected to reduce their effective population sizes, increase genetic drift, and erode local levels of genetic diversity (Brown & Knowles, 2012; Carnaval, Hickerson, Haddad, Rodrigues, & Moritz, 2009; Yannic et al., 2014). However, populations from climatically unstable areas can recurrently go extinct and be recolonized by immigrants from multiple source populations, which can increase local levels of genetic diversity via admixture (Ortego, Gugger, & Sork, 2015; Petit et al., 2003). Thus, the stability of climatically suitable habitats can impact patterns of genetic diversity and admixture in opposite directions, a possibility that has been generally overlooked (Ortego, Gugger, et al., 2015). Beyond Quaternary climatic fluctuations and contemporary landscape features, much older paleogeological events such as uplifting of mountain ranges or the emergence of islands and sea corridors are also considered important factors responsible of geographical patterns of genetic differentiation in many taxa (Ceccarelli et al., 2016; Mastretta‐Yanes, Moreno‐Letelier, Pinero, Jorgensen, & Emerson, 2015; Papadopoulou, Anastasiou, Keskin, & Vogler, 2009). Although ancient geological changes are known to underlie the spatial patterns of genetic divergence found in several organisms (Abellán, Arribas, & Svenning, 2012; Cheng et al., 2016; Opell, Helweg, & Kiser, 2016; Ortego, Bonal, Cordero, & Aparicio, 2009), in many other cases the genetic signals left by paleogeological events are expected to have been totally or partially eroded as a result of gene flow promoted by subsequent landscape changes (Graham, Hendrixson, Hamilton, & Bond, 2015; Pepper et al., 2008). Thus, examining landscape configuration at different time periods can help to better understand the mechanisms by which intraspecific genetic diversity and differentiation arise and are maintained (Reilly, Corl, & Wake, 2015).

The mountainous area of southeast Iberia has undergone remarkable geological changes that have shaped the complex biogeographical history of the region. Geological reconstructions based on stratigraphic and sedimentary data show that the emergence of mountain chains in the Tortonian (*c*. 12 Mya) configured a mosaic of islands (hereafter Betic Islands) at the confluence of European and African continental platforms. The rotation of the Betic Islands toward the Iberian Peninsula, in combination with sedimentation processes, resulted in their fusion to the continent and the configuration of a continuous emerged landscape that is currently conformed by the Prebetic, Penibetic, and Subbetic mountain ranges of southeast Iberia (Braga, Martín, & Quesada, 2003; Braga et al., 2010; Martín, Braga, Aguirre, & Puga‐Bernabéu, 2009). Furthermore, this area is also the southernmost limit of the influence of Quaternary glaciations (*c*. 2–0.04 Mya) in Europe, during which vast portions of land were free of permanent ice at elevations below 2,500 m.a.s.l. (Hughes & Woodward, 2008) and constituted an important refugium for biota from temperate habitats (Hewitt, 2000). The magnitude and complexity of these paleogeological and climate events are considered the most important engines of diversification and genetic structuring of many taxa in the region (Andújar, Gómez‐Zurita, Rasplus, & Serrano, 2012; Faille, Andujar, Fadrique, & Ribera, 2014; Fromhage, Vences, & Veith, 2004). For all these reasons, southeast Iberia is an ideal template for testing the combined effects of ancient and more contemporary climate and landscape changes on spatial patterns of genetic diversity and structure of local populations (Faille et al., 2014).

**Figure 1:**
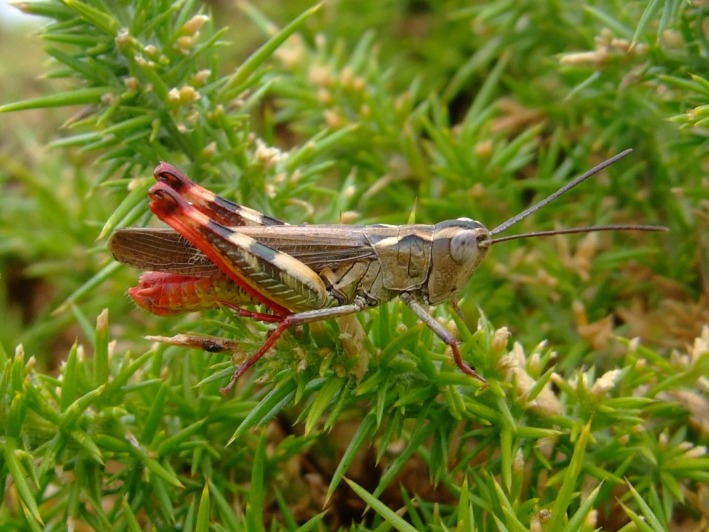
Scrub‐legume grasshopper (*Chorthippus binotatus binotatus*), the study organism. The photography shows a male specimen on a legume host plant of the genus *Ulex* (tribe *Genisteae*). Photography by Víctor Noguerales

The scrub‐legume grasshopper (*Chorthippus binotatus binotatus* Charpentier, 1825) (Orthoptera: Acrididae) is a winged Orthoptera with a 1‐year generation time (Figure 1; Defaut, 2011). This species is primarily distributed in montane regions from southwest Europe, including France and the Iberian Peninsula (Defaut, 2011). The scrub‐legume grasshopper is an oligophagous species that exclusively feeds on some scrub‐legume taxa from the tribe *Genisteae* (Defaut, 2011). In southeast Iberia, the host plants (primarily *Erinacea anthyllis* and, more occasionally, *Echinospartum boissieri*,* Genista versicolor,* and *Ulex parviflorus*) form scattered vegetation patches located at moderate to high elevations (>1,200 m.a.s.l.). This fact restricts the distribution of the scrub‐legume grasshopper to the different mountain ranges of the region (Prebetic, Penibetic and Subbetic systems) (Defaut, 2011; Table S1). Thus, the narrow ecological requirements of the scrub‐legume grasshopper, the patchy distribution of its host plants, and the limited dispersal abilities of the species (low flying capacity; V.N., P.J.C and J.O., pers. obs.) have resulted in most of its populations from southeast Iberia being currently highly fragmented and separated by extensive lowlands of unsuitable habitats (Defaut, 2011).

Here, we used the scrub‐legume grasshopper as model system to analyze the contribution of contemporary (present‐day topography and distribution of climatically suitable habitats) and historical (paleoclimate‐based distribution of suitable habitats and Late Miocene paleogeography) factors on shaping spatial patterns of genetic diversity and structure across the populations of the species from southeast Iberia. In particular, we first (1) examined geographical patterns of genetic structure and employed an approximate Bayesian computation (ABC) framework to compare different plausible scenarios of population divergence (Beaumont, 2010; Cornuet et al., 2014). Second, we (2) applied circuit theory to test whether observed patterns of genetic differentiation are explained by a comprehensive suite of isolation‐by‐resistance (IBR) scenarios (McRae, 2006; McRae & Beier, 2007), including paleogeography at different time periods since Late Miocene (*c*. 12.0–7.0 Mya; Martín et al., 2009), current and last glacial maximum (LGM, *c*. 21 Kya) climate suitability and stability, and contemporary topographic complexity (TC). Finally, we (3) tested the hypothesis predicting more genetic diversity in populations from areas with high past and present climate suitability and stability since the LGM.

## 2 Material and Methods

### 2.1 Population sampling

**Figure 2:**
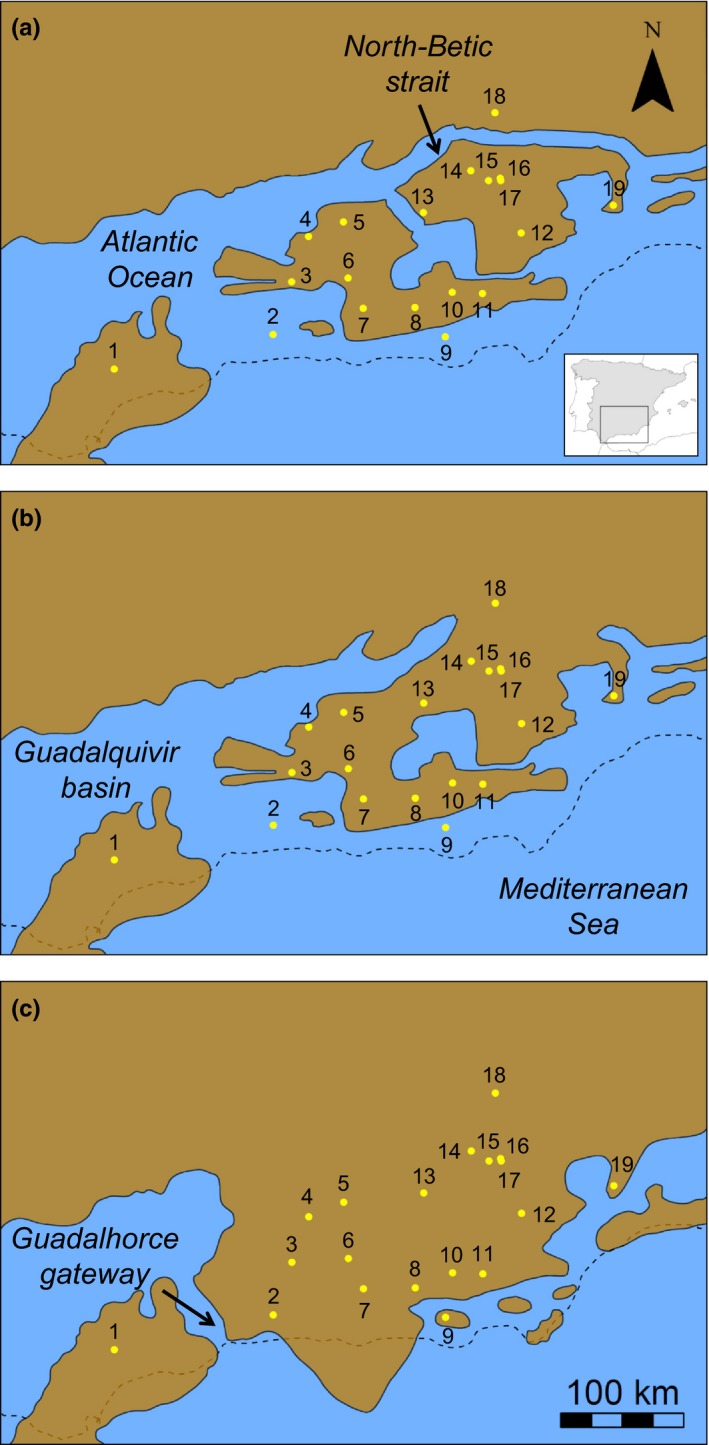
Paleogeographic maps showing the spatial configuration of emerged lands in the study area during the (a) Early Tortonian (*c*. 12.0–11.6 Mya), (b) Late Tortonian (*c*. 8.0–7.3 Mya), and (c) Earliest Messinian (*c*. 7.2–7.0 Mya) according to Martín et al. (2009). Yellow dots indicate the location of sampled populations (number codes as in Table S1). Dashed lines represent continental limits in the present. Inset map from panel (a) shows the location of our study area within the Iberian Peninsula

**Figure 3:**
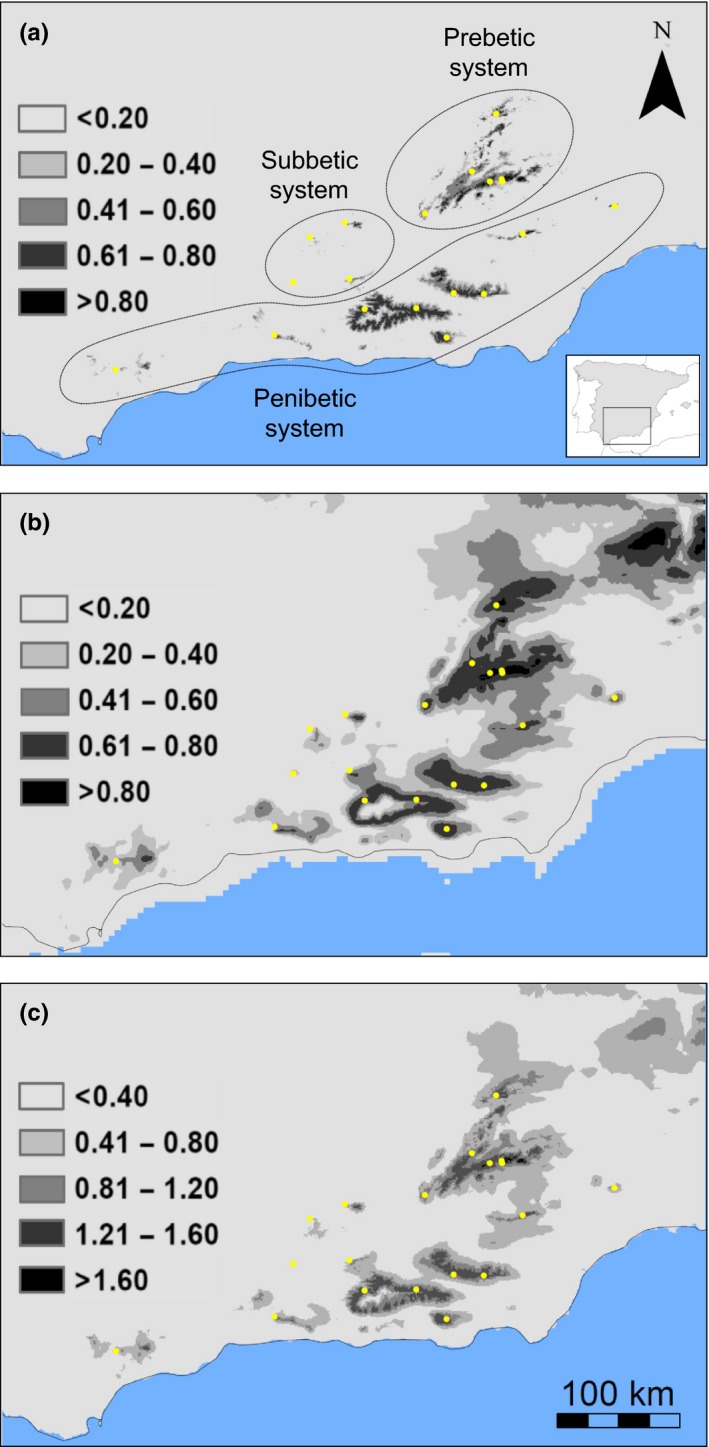
Climate niche modeling for the scrub‐legume grasshopper in southeast Iberia for (a) the present and (b) the last glacial maximum (LGM,* c*. 21 Kya). Panel (c) shows climate stability estimated as the sum of pixel values of current and LGM climate suitability maps. The LGM maps represent the average climate suitability index of the projections obtained from CCSM and MIROC climate models. Gray scales refer to climate suitability (range: 0–1) and climate stability (range: 0–2), with increasingly darker shades of gray indicating increasing climate suitability and stability. Inset map from panel (a) shows the location of our study area within the Iberian Peninsula

**Figure 4:**
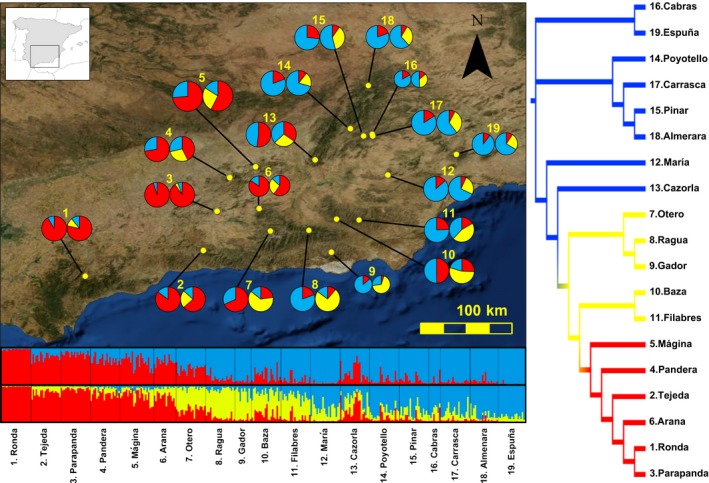
Sampling sites of scrub‐legume grasshoppers and genetic structure based on Bayesian clustering analyses. Pie charts on the map represent the genetic assignments for each sampling population according to structure analyses. For each population, left and right pie charts represent the admixture proportions considering *K *=* *2 and *K *=* *3, respectively. Circle size is proportional to the number of genotyped individuals in each population. Code numbers are described in Table S1. On the bottom, barplots represent the assignment of individuals to each genetic group according to tess analyses considering *K *=* *2 (top) and *K *=* *3 (bottom). Each individual corresponds to a vertical bar, which is partitioned into *K*‐colored segments that represent the individual's probability of belonging to the cluster with that color. Vertical black lines separate individuals from different populations. On the right, neighbor‐joining tree based on Cavalli‐Sforza and Edwards chord distances. Colors are according to structure analyses based on *K *=* *3. Inset map shows the location of our study area within the Iberian Peninsula

In 2012 and 2013, we collected 354 individuals from 19 populations of scrub‐legume grasshopper from southeast Iberia (~80,000 km^2^) (Table S1; Figures 2, 3, 4). Our sampling included populations from all mountain ranges in the region (Prebetic, Penibetic, and Subbetic ranges) and covered the entire elevation range of the scrub‐legume grasshopper in the study area (958–2,314 m.a.s.l.; Table S1). This allowed us to sample populations from different habitats such as alpine and Mediterranean scrub‐legume formations. Specimens were collected using a butterfly net, and the whole body was preserved in 2‐ml vials with 96% ethanol and stored at –20°C until needed for DNA extraction. Our sampling was performed under licenses from the “Junta de Comunidades de Castilla‐La Mancha,” “Junta de Andalucía,” and “Gobierno de la Región de Murcia.” Population codes and more information on sampling sites are presented in Table S1.

### 2.2 Microsatellite genotyping and basic genetic statistics

We extracted genomic DNA from a hind leg of each individual using a salt extraction protocol (Aljanabi & Martinez, 1997). Each individual was genotyped at 18 species‐specific microsatellites markers (Basiita et al., 2016). All microsatellite markers were polymorphic in all populations, and the most common alleles were shared across all populations. We performed PCRs and genotyping following the procedure described in Ortego, Aguirre, Noguerales, and Cordero (2015) and Basiita et al. (2016). We tested for deviations from Hardy–Weinberg equilibrium, linkage disequilibrium (LD), and the presence of null alleles as described in Noguerales, Cordero, and Ortego (2016). Two loci (Cbin16 and Cbin36) were discarded from all downstream analyses because of HW disequilibrium in all populations and the presence of null alleles. We did not find evidence for LD between any pair of loci in any sampling population after sequential Bonferroni corrections (Rice, 1989).

### 2.3 Analyses of genetic structure

We estimated population genetic differentiation calculating *F*_ST_ values between all pairs of sampling populations. Significance of genetic differentiation between all pairs of populations was tested with Fisher's exact tests after 10,000 permutations using arlequin 3.5 (Excoffier & Lischer, 2010). *p*‐Values were corrected using a sequential Bonferroni adjustment (Rice, 1989). Due to the frequent presence of null alleles in Orthoptera (Keller, Holderegger, & van Strien, 2013), we also calculated pairwise *F*_ST_ values corrected for null alleles (*F*_ST_NA) using the so‐called ENA method implemented in the program freeNA (Chapuis & Estoup, 2007).

We inferred genetic structure using Bayesian clustering analyses in structure 2.3.3 (Falush, Stephens, & Pritchard, 2003; Pritchard, Stephens, & Donnelly, 2000). We considered correlated allele frequencies and an admixture model without prior information on population origin. We performed 10 independent runs for each value of assumed number of genetic clusters (*K *=* *1–12) with a burn‐in period of 200,000 steps and a run length of 1,000,000 Markov chain Monte Carlo cycles. The number of genetic clusters (*K*) best fitting the data set was defined using log probabilities [Pr(*X*|*K*)] (Pritchard et al., 2000) and the Δ*K* method (Evanno, Regnaut, & Goudet, 2005). We used the Greedy algorithm in the program clumpp 1.1.2 (Jakobsson & Rosenberg, 2007) to align replicated runs and average individual assignment probabilities for the most likely *K* values. Finally, we used distruct 1.1 (Rosenberg, 2004) to produce bar plots displaying probabilities of individual membership to each inferred genetic cluster.

We also examined the spatial genetic structure considering geographical coordinates of sampling sites as a priori information in the Bayesian clustering method implemented in tess 2.3.1 (Chen, Durand, Forbes, & Francois, 2007; Durand, Jay, Gaggiotti, & François, 2009). We used the conditional autoregressive (CAR) Gaussian model of admixture with a linear trend surface, updating the spatial interaction parameter (ψ), initially set to the default value 0.99. The variance term (initially set to 1) permitted to update during the course of runs. CAR model was chosen in order to avoid overestimation of the most likely *K* in the presence of genetic clines (François & Durand, 2010; Guillot, 2009). We ran 20 independent replicates for each value of *K *=* *2–12 using 50,000 sweeps of which 10,000 were used as burn‐in period. The best supported number of genetic clusters (*K*) was estimated using the deviance information criterion (DIC) values and stabilization of the *Q* matrix of posterior probabilities (Chen et al., 2007; Gao, Bryc, & Bustamante, 2011). For each *K*_MAX_‐value considered, we conducted 180 additional replicate runs up to a total of 200 replicates. We used the 10 runs with the lowest DIC values to align and average individual assignment probabilities with clumpp before being represented using distruct as indicated above for structure analyses.

Complementarily, we constructed a phylogenetic tree to visualize the genetic relationships between all populations. We used the program populations 1.2.31 (Langella, 1999) to obtain a neighbor‐joining tree based on pairwise Cavalli‐Sforza and Edwards (*D*_*c*_) genetic distances (Cavalli‐Sforza & Edwards, 1967). Finally, we carried out analyses of molecular variance (amovas) to examine the partitioning of the genetic variation among and within regions and populations as defined by five population grouping hypotheses. Populations were pooled according to their historical location in the three different paleogeographical Late Miocene scenarios (see Figure 2 and section “Landscape genetic analyses”) and their current location in the main mountain ranges of the region (Prebetic, Penibetic, and Subbetic systems; see Table S1 and Figure 3a). Additionally, we tested the grouping scheme used for ABC analyses (see next section). amovas were performed in arlequin 3.5 (Excoffier & Lischer, 2010), and the significance of the variance components was tested using 10,000 permutations of the original data.

### 2.4 Approximate Bayesian computation

**Figure 5:**
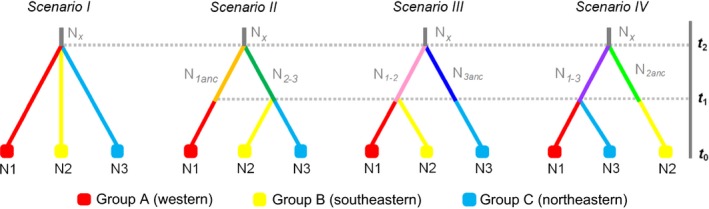
Scenarios compared using an approximate Bayesian computation (ABC) approach (*t*# represents time in number of generations; *N*# represents effective population sizes during each time period)

In order to infer the evolutionary and demographic history of the scrub‐legume grasshopper in the region, we compared four plausible scenarios of population divergence using an ABC approach (Beaumont, 2010). To simplify the analyses, we defined three main groups (groups A, B, and C) of populations by pooling sampling sites according to their geographical location and the results from amovas (Table S2) and Bayesian clustering analyses (structure and tess) (e.g., Tsuda, Nakao, Ide, & Tsumura, 2015). Note that although *K *=* *2 was the most supported clustering solution for both structure and tess analyses, *K *=* *3 revealed further hierarchical genetic substructure with geographical coherence (see the “3” section and Figure S1). In group A, we included populations 1–6 (western populations); group B, 7–12 (southeastern populations); and group C, 13–19 (northeastern populations) (see Figures 4 and 5). The topology of each scenario was designed considering the connectivity of populations according to Bayesian clustering analyses (Figure 4 and Figure S1c). The scenarios tested were the following: (1) Scenario I, null model: The three groups diverged simultaneously; (2) Scenario II, sequential splitting model from west to east: Group A split from group B and C at *t*_2_, and these two groups subsequently split at *t*_1_; (3) Scenario III, sequential splitting model from east to west: Group C split from groups A and B at *t*_2_, and these two groups split at *t*_1_; (4) Scenario IV, splitting model from central to peripheral populations: Group B split from groups A and C at *t*_2_, and these two groups subsequently split at *t*_1_ (Figure 5).

We conducted all the computations using diyabc 2.0.4 (Cornuet et al., 2014). We generated 3 millions of simulated data sets per scenario considering a generalized mutation model and no single‐nucleotide indels (Table S3). The summary statistics (SS) used in ABC analyses are described in Table S3. We performed pre‐evaluation of scenarios and prior distributions in diyabc to adjust the priors of *N*_e_ and *t* to their most appropriate values (see Table S3), assuming a uniform prior probability distribution for them. To avoid biases in parameter estimates, we selected the subset of seven microsatellites markers with lower frequency of null alleles as estimated in the program freeNA. Selection of the most probable scenario, confidence in scenario choice (type I and II errors), model checking, and estimation of the posterior distribution of all parameters under the best supported model were performed as described in Ortego, Noguerales, Gugger, and Sork (2015).

### 2.5 Landscape genetic analyses

We applied circuit theory (McRae, 2006; McRae & Beier, 2007) and a multiple matrix regression with randomization (MMRR) approach (Wang, 2013) to examine the relative contribution of a suite of IBR scenarios to explain patterns of genetic differentiation in our study populations. Specifically, we tested nine different hypothetical scenarios of population connectivity, which included (1) three paleogeographic scenarios defined by the spatial configuration of emerged lands at different time periods (Early Tortonian, Late Tortonian, and Earliest Messinian); (2) three scenarios based on the distribution of climatically suitable habitats since the LGM (current climate suitability, LGM climate suitability, and climate suitability stability since the LGM); (3) a scenario of population connectivity defined by contemporary TC; (4) an isolation‐by‐distance (IBD) scenario representing the geographical distance between each pair of populations. Below we describe in detail the methods followed to generate these scenarios and test their relative contribution to contemporary patterns of genetic differentiation.

#### 2.5.1 Paleogeographic scenarios

To test the possible effect of the complex geological history of the study region on contemporary patterns of genetic differentiation, we considered three paleogeographic scenarios: Early Tortonian (12.0–11.6 Mya), Late Tortonian (8.0–7.3 Mya), and Earliest Messinian (7.2–7.0 Mya) (see Figure 2). We used arcgis 10.0 (ESRI, Redlands, CA, USA) to create vector layers for emerged lands based on geological models from Martín et al. (2009). Then, we transformed vectors layers for each scenario into raster maps with a 30 arc‐sec (*c*. 1 km) resolution that were finally used as inputs in circuitscape (McRae, 2006; McRae & Beier, 2007) (see below for details on circuitscape analyses).

#### 2.5.2 Climatic suitability scenarios

We modeled the potential climate distribution of scrub‐legume grasshopper at different time periods to investigate whether the spatial distribution of climatically suitability habitats are relevant factors shaping observed patterns of genetic differentiation in the study populations. For this purpose, we built a climate niche model (CNM) using the maximum entropy presence‐only algorithm implemented in maxent 3.3.3 (Phillips, Anderson, & Schapire, 2006; Phillips & Dudik, 2008) based on current climate. We used a total of 85 occurrence points obtained from the Global Biodiversity Information Facility, the literature (Defaut, 2011), and our own sampling. To construct the models, we used the 19 bioclimatic variables available in WorldClim and downloaded at 30 arc‐sec (*c*. 1 km) resolution (Hijmans, Cameron, Parra, Jones, & Jarvis, 2005). Variables retained in the final models were selected following several complementary criteria (Vega et al., 2010). At first, we used ENMtools (Warren, Glor, & Turelli, 2010) to examine colinearity among variables, in order to retain a single layer among those with a high Pearson correlation coefficient (*r *>* *.85). Then, we used the Jackknife of regularized training gain procedure implemented in maxent to retain the variables with the maximum contribution to the model. We discarded the worst and highest correlated predictors among the whole set of variables, conducted a new model with the remaining variables, and repeated this backward process until the final model only retained the best explanatory and less correlated variables (Vega et al., 2010). Model evaluation statistics were produced from 10 cross‐validation replicate model runs.

To obtain the distribution of scrub‐legume grasshopper during the LGM (LGM, *c*. 21 Kya), we projected contemporary species–climate relationships to the LGM using two atmospheric circulation models: the Community Climate System Model (CCSM3; Collins et al., 2006) and the Model for Interdisciplinary Research on Climate (MIROC 3.2; Hasumi & Emori, 2004) from the Paleoclimate Modelling Intercomparison Project Phase II (PMIP2; Braconnot et al., 2007). LGM layers were downloaded from WorldClim at 2.5 arc‐min and interpolated to 30 arc‐sec resolution. To reduce the level of uncertainty arising from different past projections, we averaged climate suitability scores from projections based on CCSM and MIROC models to obtain a consensus LGM map of climatically suitable areas. In addition, we summed current and LGM climate suitability layers to generate a map of climate suitability stability, with pixel values ranging from 0 (minimum climate suitability in both periods) to 2 (maximum climate suitability in both periods). All GIS calculations were conducted in arcgis10.0. Finally, current, LGM, and stability climate suitability raster maps were used as inputs in circuitscape to calculate IBR distance matrices (see below for details).

#### 2.5.3 Contemporary topographic complexity scenario

We investigated the role of contemporary TC as a potential factor shaping patterns of genetic differentiation in our study populations. We calculated the surface ratio index for each cell from a present‐day digital elevation model using “DEM surface tools” (Jenness, 2013) in arcgis 10.0. Surface ratio is an index of TC, with values close to one indicating flat areas and values higher than one indicating a more abrupt relief with deeper slopes (Jenness, 2004). Calculations were conducted on a 90‐m resolution digital elevation model from NASA Shuttle Radar Topographic Mission (SRTM Digital Elevation Data). Although no information is available on the dispersal distance and home range of the study species, the high resolution of the digital elevation model is expected to capture well the TC relevant for a medium‐size grasshopper with a suspected low dispersal ability. The final raster map was transformed to 30 arc‐sec (*c*. 1 km) resolution and used as input in circuitscape (see below for details).

#### 2.5.4 circuitscape analyses

We used circuitscape 4.0 (McRae, 2006; McRae & Beier, 2007) to calculate resistance distance matrices between all pairs of populations considering an eight‐neighbor cell connection scheme. The raster layers generated for the nine different scenarios of population connectivity were used as inputs in circuitscape. For the three paleogeographic scenarios, the raster layers included two element classes: “emerged land” and “sea water.” We considered that “sea water” was the main landscape feature limiting the dispersal of terrestrial fauna in the study area during these periods. We generated different IBR scenarios assigning different resistance values to “sea water” (10, 50, 100, 500, 1,000, 10,000), an approach that allowed us to identify the optimal ratio of landscape resistance between both landscape elements that best fit our data on genetic differentiation (e.g., Andrew, Ostevik, Ebert, & Rieseberg, 2012; Ortego, Aguirre, et al., 2015). To test the effect of IBD, we calculated pairwise resistance distances on a completely “flat” landscape based on a raster layer in which all cells had an equal value (conductance = 1). This IBD resistance model is expected to yield similar results than a matrix of Euclidean geographical distances, but it is more appropriate for comparison with others competing models also generated with circuitscape (Velo‐Antón, Parra, Parra‐Olea, & Zamudio, 2013).

#### 2.5.5 Statistical analyses

We used a MMRR approach to examine the relative contribution of all IBR and IBD scenarios to explain patterns of genetic differentiation in our study populations (Wang, 2013). We tested the two matrices of genetic differentiation (*F*_ST_ and *F*_ST_NA) against all pairwise resistance distance matrices representing the nine different IBR/IBD scenarios. We used a backward procedure to select final models, eliminating nonsignificant variables from an initial full model including all explanatory predictors. We tested the significance of the remaining variables again until no additional term reached significance (Noguerales et al., 2016; Ortego, Gugger,et al., 2015).

### 2.6 Analyses of genetic diversity and admixture

Allelic richness (*A*_R_) standardized for sample size was calculated for each population using hp‐rare (Kalinowski, 2005). We estimated the genetic admixture of populations using a genetic admixture index (*G*_ADMIX_) obtained from the probabilities of population membership to each genetic cluster inferred by structure analyses (Ortego, Gugger, et al., 2015). This index was designed to standardize the degree of genetic admixture across populations with different probabilities of membership to different genetic clusters (Ortego, Gugger, et al., 2015), and its advantages and potential caveats are those inherent to structure analyses (Falush et al., 2003; Pritchard et al., 2000). *G*_ADMIX_ ranges from 0 (indicating no admixture, i.e., genetically pure populations assigned to a single genetic cluster) to 1 (indicating maximum admixture, i.e., genetically admixed populations with an equal probability of membership to each inferred genetic cluster). We used generalized linear models (GLMs, using a Gaussian error distribution and an identity link function) and an information‐theoretic model selection approach to analyze *A*_R_ and *G*_ADMIX_ (Burnham & Anderson, 2002). Models for *A*_R_ included as independent variables current climate suitability (*HS*_CUR_), LGM climate suitability (*HS*_LGM_), and climate suitability stability (*HS*_STA_). Models for *G*_ADMIX_ included *HS*_STA_ as independent variable. Longitude and latitude were included as additional covariates in models for both *A*_R_ and *G*_ADMIX_ to take in account possible geographical clines of genetic diversity and admixture (e.g., Guo, 2012). We calculated average *HS*_CUR_, *HS*_LGM_, and *HS*_STA_ with arcmap 10.0 at different spatial scales using buffers of 1, 10, and 100 km^2^ around sampling locations. Given that the precision of *A*_R_ and *G*_ADMIX_ estimates may differ among populations due to differences in sample sizes, we used a weighted least square method where weight equals the sample size for each studied population. GLMs were built in the R package lme4 (Bates, Maechler, Bolker, & Walker, 2015; R Core Team, 2015) and model selection and averaging were performed using the R package muMin (Barton, 2015) as detailed in Noguerales, Traba, Mata, and Morales (2015) and Ortego, Aguirre, et al. (2015).

## 3 Results

### 3.1 Population genetic structure

We found that most pairs of populations were genetically differentiated. In particular, 139 of 171 pairwise *F*_ST_ values (~81%) were significantly higher than zero after sequential Bonferroni correction (Table S4). Significant pairwise *F*_ST_ values ranged from .025 to .164, whereas pairwise *F*_ST_NA‐values were slightly lower and ranged from .008 to .144. Pairwise *F*_ST_ and *F*_ST_NA‐values were highly correlated (Mantel *r *=* *.993; *p *<* *.001). The populations from Ronda, Parapanda, and Tejeda, located at the westernmost portion of the study area, exhibited the highest levels of genetic differentiation with the rest of populations. Analyses in structure showed a best supported number of clusters for *K *=* *2 according to the Δ*K* method. The first cluster included the Western populations, whereas the second cluster included the remaining populations located in the east part of the study area. However, log probabilities [Ln Pr (X|*K*)] steadily increased from *K *=* *2 to *K *=* *5 (Figure S1a). Individual assignment probabilities to a certain genetic cluster were moderately high up to *K *= 5 and the spatial distribution of genetic variation exhibited geographical consistency, but most populations showed a considerable degree of genetic admixture (Figure 4, Figure S1c). Genetic clustering analyses in tess resulted in an optimal *K *=* *4 according to the DIC criterion (Figure S1b), but one of the inferred clusters represented a “ghost cluster” with no individual assigned to it (see Chen et al., 2007; Guillot, Estoup, Mortier, & Cosson, 2005). When *K *=* *3 was considered, the first cluster included the Western populations, the second cluster included the southeastern populations, and the third cluster included the northeastern populations of the study area (Figure 4). tess and structure analyses yielded similar results for *K *=* *2 and *K* = 3 (Figure S1c). The result of the neighbor‐joining tree based on Cavalli‐Sforza and Edwards chord distances (*D*_c_) was also congruent with the results from Bayesian clustering analyses (Figure 4). Finally, amova analyses indicated most genetic variance was attributed to differences within populations (>90%, for all grouping hypotheses; Table S2). The population grouping hypothesis that explained the highest percentage of total variation attributed to differences among groups was the one used for ABC analyses (Table S2).

### 3.2 Approximate Bayesian computation

The scenario considering sequential population divergence from west to east (scenario II) had the highest posterior probability based on both direct and logistic regression‐based estimates, and its 95% confidence interval did not overlap with those obtained for others scenarios that showed much lower support (Table 1). Observed data fell within simulated data (all SS *P*s > .2) for scenario II, suggesting good model fit. Type I and II errors were .394 and .372, respectively, and RMAE values were moderate in most cases (Table 1). Considering the 1‐year generation time of scrub‐legume grasshopper (Defaut, 2011), the western genetic group (group A) diverged from the southeastern and northeastern groups (groups B and C, respectively) ~215,000 years ago (*t*_2_) (95% CI: 67,600–342,000 years ago), whereas these two groups split ~42,000 years ago (*t*_1_) (95% CI: 5,540–165,000 years ago) (Table 2). Assuming a constant mutation rate, the posterior estimates of effective populations sizes (*N*_e_) indicated no important demographic changes after the different splitting events (Table 2).

**Table 1:** Posterior probability for each of the four tested scenarios and 95% confidence intervals (CI) based on the weighted polychotomous logistic regression approach for approximate Bayesian computation (ABC) analyses. Type I and type II errors for the best supported scenario (in bold) are indicated

Scenario	Posterior probability	95% CI	Type I error	Type II error
I	.0230	[0.0222**–**0.0253]		
**II**	**.9618**	**[0.9595–0.9640]**	**.394**	**.372**
III	.0059	[0.0054**–**0.0064]		
IV	.0085	[0.0078**–**0.0093]		

**Table 2:** Posterior parameter estimates (median and 95% confidence intervals) for the best supported scenario (scenario 2, see Figure 5). Estimates are based on 1% of simulated data sets closest to the observed values. Relative median absolute errors (RMAE) based on 500 pseudo‐observed data sets are also indicated for each parameter

Parameter	Median	*q*_0.025_	*q*_0.975_	RMAE
N1	520,000	194,000	736,000	.279
N2	407,000	113,000	715,000	.246
N3	600,000	277,000	740,000	.258
N_1anc_	303,000	39,800	690,000	.394
N_2‐3_	345,000	46,600	695,000	.372
N_x_	57,000	24,800	541,000	.384
*t*_1_	42,700	5,540	165,000	.404
*t*_2_	215,000	67,600	342,000	.213
μ	7.75 × 10^−6^	4.24 × 10^−6^	2.85 × 10^−5^	.357

N1, effective population size of group A; N2, effective population size of group B; N3, effective population size of group C; N_1anc_, effective population size of the ancestral group A; N_2‐3_, effective population size of the ancestral groups B‐C; N_x_, effective population size of the most ancestral population, *t*_1_, time (in generations = years) to the most recent divergence event; *t*_2_, time (in generations = years) to the most ancient divergence event (see scenarios in Figure 5); μ, mean mutation rate.

### 3.3 Climate niche modeling

The variables included in the final CNM were temperature seasonality (BIO4), mean temperature of the driest quarter (BIO9), precipitation of the driest month (BIO14), and precipitation of the warmest quarter (BIO18). This model had a very high value of area under the curve (AUC; 0.982 ± 0.007), indicating overall good performance. The predicted distribution of scrub‐legume grasshopper in the present (Figure 3a) is consistent with its observed fragmented distribution. The distribution of the species was more extensive and populations were better connected during the LGM than at present time (Figure 3b). However, populations located in the western portion of our study area (corresponding with group A in ABC analyses) have remained highly isolated during both the LGM and the present.

### 3.4 Landscape genetic analyses

**Figure 6:**
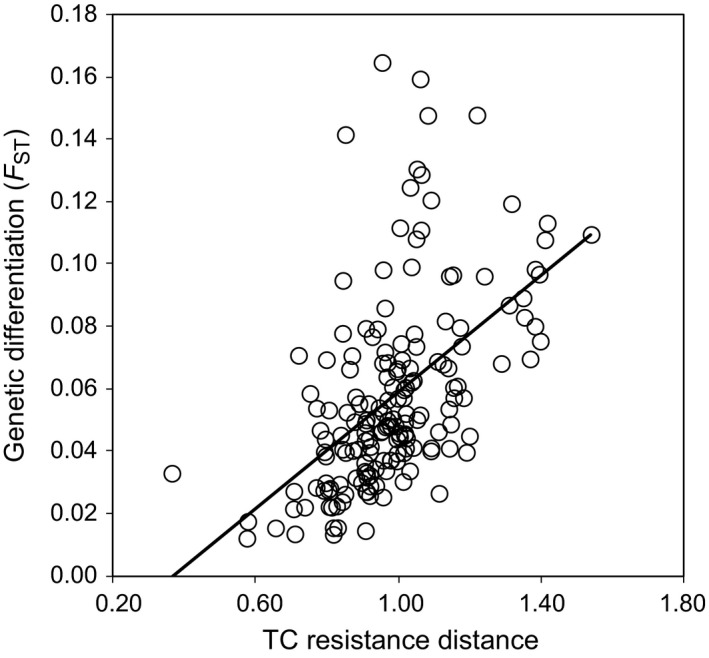
Relationship between genetic differentiation (*F*_ST_) and resistance distances calculated using circuitscape on the basis of contemporary topographic complexity (TC)

Tejeda and Ronda populations are currently located in areas that were not likely to form emerged lands during Early and Late Tortonian, and for this reason, they were excluded from landscape genetic analyses. Thus, we performed MMRR analyses using the 17 populations presumably located on permanently emerged lands since the late Miocene in order to make our landscape genetic analyses comparable across all tested scenarios and time periods. Considering these 17 populations, only resistance distances based on contemporary TC and IBD were significantly associated with genetic differentiation (*P*s < .006) (Table 3). However, only TC was retained into the final model (β = .826, *t *=* *7.56, *p *=* *.004) (Figure 6). Analyses based on *F*_ST_NA gave similar results (Table 3), but models had slightly lower values of *r* ^2^. Analyses considering all populations (*n *=* *19) yielded qualitatively analogous results (data not shown).

**Table 3:** Results of univariate matrix regressions with randomization (MMRR) for genetic differentiation [*F*_ST_ and *F*_ST_ corrected for null alleles (*F*_ST_NA)] in relation to different isolation‐by‐resistance (IBR) scenarios: geographical distance (IBD), contemporary topographic complexity (TC), current climate suitability (HS_CUR_), last glacial maximum climate suitability (HS_LGM_), climate suitability stability (HS_STA_), and three paleogeographical models (Early Tortonian, *c*. 12.0–11.6 Mya; Late Tortonian, *c*. 8.0–7.3 Mya; Earliest Messinian, *c*. 7.2–7.0 Mya)

Model	*F*_ST_				*F*_ST_NA			
	*r* ^2^	β	*t*	*p*	*r* ^2^	β	*t*	*p*
IBD	.298	.824	7.543	.004	.273	.811	7.097	.011
TC	.299	.826	7.561	.006	.274	.814	7.118	.021
HS_CUR_	.031	.163	2.084	.264	.055	.224	2.815	.086
HS_LGM_	.220	.446	6.16	.051	.194	.429	5.662	.064
HS_STA_	.210	.432	5.978	.053	.185	.417	5.521	.062
Early Tortonian	.115	.320	4.180	.063	.096	.301	3.780	.055
Late Tortonian	.101	.296	3.886	.066	.084	.278	3.522	.052
Earliest Messinian	.088	.278	3.612	.065	.072	.258	3.237	.062

For paleogeographical models, we considered high resistance values for sea water (=100) and low for emerged lands (=1). Table shows the results based on the 17 populations presumably located on permanently emerged lands since the Late Miocene.

### 3.5 Analyses of genetic diversity and admixture

*G*_ADMIX [*K* = *i*, 5]_ indexes obtained considering different *K* values were highly correlated among them (all *r > *.598, all *P*s < .007). Population genetic admixture based on any *G*_ADMIX [*K *= *i*, 5]_ index and *A*_R_ were also correlated (all *r > *.451, all *P*s < .050). Model selection results showed that *A*_R_ was not significantly associated with longitude, latitude, or *HS*_CUR_, *HS*_LGM_, or *HS*_STA_ at any analyzed spatial scale (all unconditional 95% CIs of the predictors crossed zero; Table S5). Likewise, population genetic admixture (based on any *G*_ADMIX [*K *= *i*, 5]_ index) was not significantly associated with longitude, latitude, or *HS*_STA_ at any analyzed spatial scale (all unconditional 95% CIs of the predictors crossed zero; Table S6).

## 4 Discussion

Assuming ecological niche stability through time, our niche model revealed a moderate shift in the distribution of climatically suitable habitats for the scrub‐legume grasshopper in southeast Iberia during the last 21,000 years (Nogués‐Bravo, 2009). Climate niche modeling indicated that the potential distribution of the species is more fragmented in the present than during the LGM, a pattern congruent with the increased population connectivity during glacial periods inferred for many other montane species from temperate regions (Blanco‐Pastor, Fernández‐Mazuecos, & Vargas, 2013; Velo‐Antón et al., 2013). Continuous climatically suitable habitats connected the southern foothills of the Prebetic mountain range and the eastern portion of the Penibetic system during the LGM, an area where the species is not present today as verified by our own surveys. Our CNM also outlined the isolation of the Western populations since LGM, which has probably contributed to shape their strong genetic differentiation with the rest of the populations within the study area. The isolation of the Western populations could have occurred during the last interglacial (LIG) period (*c*. 120,000–140,000 years ago), when the scrub‐legume grasshopper probably showed a distribution similar to that in the present. We did not find support for the hypothesis predicting more genetic diversity in populations from areas with higher past and current climate suitability and stability since the LGM. Unlike other organisms from temperate regions whose distributions were confined to isolated refugia during glacial or interglacial periods (Homburg et al., 2013), the CNM for the scrub‐legume grasshopper indicate a considerable stability of climate suitability since LGM in most of the study area. Thus, the lack of major range shifts and geographically restricted climate refugia may explain why genetic diversity and admixture are decoupled from local stability of climatically suitable habitats (Ortego, Gugger, et al., 2015; Petit et al., 2003; Yannic et al., 2014).

Populations of scrub‐legume grasshopper showed higher levels of genetic differentiation than those observed at similar spatial scales in generalist grasshoppers (Blanchet, Lecoq, Sword, Berthier, et al., 2012; Blanchet, Lecoq, Sword, Pages, et al., 2012; Keller, Holderegger, et al., 2013; Keller, van Strien, et al., 2013; Ortego, Aguirre, et al., 2015; Wiesner et al., 2011), but lower than in populations of specialist Orthoptera inhabiting highly fragmented habitats (Ortego, Aguirre, & Cordero, 2012; Streiff, Audiot, Foucart, Lecoq, & Rasplus, 2006). The highest pairwise *F*_ST_ values were found in comparisons involving the Western and easternmost populations of the Penibetic system, which are located at the extremes of the species distribution range in the study area and that seem to have remained poorly connected according to our CNM (Figure 3). Despite the relatively small size of the study area (the most distant populations are separated by <400 km), the neighbor‐joining tree and Bayesian clustering analyses revealed a moderate degree of spatial genetic structure. The three genetic clusters found in our study populations spanned different mountain systems: western Penibetic and Subbetic (western cluster), central Penibetic (southeastern cluster), and easternmost Penibetic and Prebetic ranges (northeastern cluster). Similar geographical patterns of genetic structure have been found in other codistributed organisms from the region, which has been interpreted as a result of long‐term isolation during the Pleistocene of populations inhabiting different mountain ranges (Albert, Zardoya, & García‐París, 2007; Dias et al., 2015). Our Bayesian clustering analyses also showed a relatively high degree of genetic admixture that was more evident in populations located at the core of our study area (southern Prebetic and eastern Penibetic systems), which may reflect their higher connectivity with the rest of the populations. ABC analyses supported a sequential splitting model in which population divergence took place from the western to the eastern portion of the study area (scenario II). The Western populations diverged from the remaining populations ~215 Kya, while the southeastern and northeastern populations split ~42.7 Kya. This result suggests that the most ancient divergence event likely took place during the interglacial period between the Riss and Mindel glaciations, whereas the most recent divergence event could have occurred during a short warm period between the LGM (*c*. 21 Kya) and the LIG (*c*. 120 Kya) (Siddall et al., 2003). Both warm periods were characterized by a generalized expansion of deciduous and Mediterranean forests and the contraction of the vegetation adapted to dry and cool climates (Sánchez Goñi et al., 2005). The distribution of shrub‐like vegetation probably retracted to higher elevations during interglacial periods, which may have promoted the isolation and progressive differentiation of the populations of scrub‐legume grasshopper. However, we must note that our estimates of divergence times should be interpreted with extreme caution given that the confidence intervals for both *t*_2_ (95% CI: 67,600–342,000 years ago) and *t*_1_ (95% CI: 5,540–165,000 years ago) were very broad. In addition, it must be also considered that diyabc does not accommodate gene flow after divergence (Cornuet et al., 2014), which is expected to underestimate divergence times (Ortego, Noguerales et al., 2015; Tsuda et al., 2015).

Our landscape genetic analyses showed that resistance distances based on contemporary TC provided the best model fit, indicating that gene flow is primarily shaped by physical features of the landscape (Wang, 2012). This suggests that the contemporary TC of southeast Iberia is playing a more important role in determining dispersal of scrub‐legume grasshoppers than the distribution of climatically suitable habitat patches. A similar result was found for the Morales grasshopper (*Chorthippus saulcyi moralesi*), an endemic taxon from the Pyrenees belonging to the same species complex than the scrub‐legume grasshopper (Noguerales et al., 2016). This is in line with several studies on other taxa finding that topography is one of the main predictors of genetic differentiation in terrestrial organisms, a fact that has been linked to the greater energetic expenditure associated with dispersal across abrupt reliefs (Benham & Witt, 2016; Castillo et al., 2014; Hemp, Grzywacz, Warchalowska‐Sliwa, & Hemp, 2016; Velo‐Antón et al., 2013). We did not find a significant relationship between genetic differentiation and resistance distances based on any climate suitability or paleogeographic scenario. The lack of association between genetic differentiation and resistance distances based on climate suitability could be explained by the fact that the observed patterns of genetic differentiation were shaped by environmental factors predating the LGM period as suggested by our ABC analyses. An alternative explanation for such lack of association could be that our CNM is not capturing well the microhabitat structure that defines the spatial configuration of corridors/barriers to dispersal in our study species (Noguerales et al., 2016). Different factors could be behind the lack of association between the spatial genetic structure and ancient geological changes: (1) The genetic signature eventually left during the Tortonian as consequence of isolation processes could have been progressively eroded over time due to post‐Messinian population connectivity and gene flow; (2) the Betic islands could have remained noncolonized by the scrub‐legume grasshopper until the Earliest Messinian (*c*. 7 Mya) due to the large distance to the mainland and/or the absence of optimal habitats given the warmer climate and the forest‐like vegetation prevailing during the Late/Middle Miocene (*c*. 23–14 Mya) (Jimenez‐Moreno, Fauquette, & Suc, 2010). Finally, (3) the high mutation rates of microsatellite markers that make them adequate to track recent and ongoing processes of genetic differentiation are expected to reduce their power to capture signatures of old demographic events such as those driven by ancient geological changes (Anderson et al., 2010; Velo‐Antón et al., 2013; Wang, 2010; Zellmer & Knowles, 2009).

Overall, this study shows that contemporary TC is the main landscape factor predicting spatial patterns of population genetic differentiation in the scrub‐legume grasshopper. Future studies analyzing the complete distribution range of the species and considering current and past distributional data for all host plant taxa can provide new insights into the consequences of ancient and contemporary landscape changes on the evolutionary and demographic history of this specialist grasshopper. Our study emphasizes the need of integrating spatial data of ancient and contemporary landscape composition to get a comprehensive view of their relative importance in explaining genetic variation of organisms inhabiting regions with a complex biogeographical history.

## Conflict of Interest

None declared.
