# Supplementary material for: Testing the role of ancient and contemporary landscapes on structuring genetic variation in a specialist grasshopper
Source: Ecol Evol. 2017 Mar 30;7(9):3110–22. doi: 10.1002/ece3.2810 (PMC5415511; doi:10.1002/ece3.2810)
Supplement: Supplementary file 1 [file ECE3-7-3110-s001.docx]

**- SUPPORTING INFORMATION -**

*Ecology and Evolution*

**Title:**

*Testing the role of ancient and contemporary landscapes on structuring genetic variation in a specialist grasshopper*

**Authors**:

Víctor Noguerales

Grupo de Investigación de la Biodiversidad Genética y Cultural,

Instituto de Investigación en Recursos Cinegéticos - IREC (CSIC, UCLM, JCCM),

Ronda de Toledo 12, E-13071 Ciudad Real, Spain.

E-mail (corresponding autor): [victor.noguerales@csic.es](mailto:victor.noguerales@csic.es)

Pedro J. Cordero

Grupo de Investigación de la Biodiversidad Genética y Cultural,

Instituto de Investigación en Recursos Cinegéticos - IREC (CSIC, UCLM, JCCM),

Ronda de Toledo 12, E-13071 Ciudad Real, Spain.

E-mail: [pedrojavier.cordero@uclm.es](mailto:pedrojavier.cordero@uclm.es)

Joaquín Ortego

Department of Integrative Ecology,

Estación Biológica de Doñana, EBD-CSIC,

Avda. Américo Vespucio s/n, E-41092 Seville, Spain.

E-mail: [joaquin.ortego@csic.es](mailto:joaquin.ortego@csic.es)

**Table S1** Geographical location, number of genotyped individuals (*N*) and population genetic variability (allelic richness standardized for sample size, *A*_R_) for the studied populations of the scrub-legume grasshopper in southeast Iberia.

| ID | Locality | Code | Mountain range | Latitude | Longitude | Elevation (m) | *N* | *A*_R_ |
| --- | --- | --- | --- | --- | --- | --- | --- | --- |
| 1 | Ronda | RON | Penibetic | 36.663990 | -5.229845 | 958 | 20 | 5.39 |
| 2 | Tejeda | TEJ | Penibetic | 36.918736 | -4.066843 | 1595 | 20 | 5.65 |
| 3 | Parapanda | PAR | Subbetic | 37.305478 | -3.929667 | 1578 | 20 | 4.55 |
| 4 | Pandera | PAN | Subbetic | 37.636927 | -3.805924 | 1512 | 20 | 5.56 |
| 5 | Mágina | MAG | Subbetic | 37.744182 | -3.549024 | 1325 | 23 | 5.61 |
| 6 | Arana | ARA | Subbetic | 37.331304 | -3.516227 | 1736 | 15 | 5.16 |
| 7 | Otero | OTE | Penibetic | 37.110127 | -3.405121 | 2314 | 20 | 5.45 |
| 8 | Ragua | RAG | Penibetic | 37.116477 | -3.026024 | 2100 | 20 | 4.99 |
| 9 | Gador | GAD | Penibetic | 36.901460 | -2.803908 | 2017 | 11 | 5.17 |
| 10 | Baza | BAZ | Penibetic | 37.227329 | -2.752059 | 1876 | 20 | 5.50 |
| 11 | Filabres | FIL | Penibetic | 37.219673 | -2.530702 | 2117 | 20 | 5.55 |
| 12 | María | MAR | Penibetic | 37.662071 | -2.246718 | 1670 | 20 | 5.70 |
| 13 | Cazorla | CAZ | Prebetic | 37.810585 | -2.962646 | 1772 | 20 | 5.64 |
| 14 | Poyotello | POY | Prebetic | 38.119515 | -2.616541 | 1600 | 20 | 5.53 |
| 15 | Pinar | PIN | Prebetic | 38.046300 | -2.485148 | 1662 | 20 | 5.99 |
| 16 | Cabras | CAB | Prebetic | 38.062612 | -2.399850 | 2038 | 8 | 5.07 |
| 17 | Carrasca | CAR | Prebetic | 38.045469 | -2.396126 | 1526 | 20 | 5.55 |
| 18 | Almenara | ALM | Prebetic | 38.543520 | -2.440236 | 1655 | 19 | 5.78 |
| 19 | Espuña | ESP | Penibetic | 37.865166 | -1.571250 | 1514 | 18 | 5.08 |

**Table S2** Results of Amovas used to test different population grouping hypotheses for scrub-legume grasshopper in southeast Iberia. NS = Not significant; * *P* < 0.05; ** *P* < 0.01; *** *P* < 0.001.

| Hypothesis | Hierarchical level | Sum of  squares | Variance  components | %  variance |
| --- | --- | --- | --- | --- |
| Early Tortonian | Among groups (four groups) | 89.06 | 0.094 | 1.53* |
| *(17 populations)* | Among populations within groups | 221.43 | 0.297 | 4.80*** |
|  | Within populations | 3655.45 | 5.811 | 93.67*** |
|  |  |  |  |  |
| Late Tortonian | Among groups (two groups) | 31.36 | 0.160 | 2.54** |
| *(17 populations)* | Among populations within groups | 279.12 | 0.338 | 5.36*** |
|  | Within populations | 3655.45 | 5.811 | 92.09*** |
|  |  |  |  |  |
| Earliest Messinian | Among groups (three groups) | 34.16 | 0.001 | 0.02 NS |
| *(19 populations)* | Among populations within groups | 310.49 | 0.358 | 5.79*** |
|  | Within populations | 4011.97 | 5.822 | 94.19*** |
|  |  |  |  |  |
| Mountain ranges | Among groups (three groups) | 77.96 | 0.097 | 1.57** |
| *(19 populations)* | Among populations within groups | 266.69 | 0.293 | 4.72*** |
|  | Within populations | 4011.97 | 5.822 | 93.71*** |
|  |  |  |  |  |
| ABC grouping | Among groups (three groups) | 126.09 | 0.208 | 3.33*** |
| *(19 populations)* | Among populations within groups | 218.56 | 0.211 | 3.39*** |
|  | Within populations | 4011.97 | 5.822 | 93.27*** |

**Table S3** Prior distributions of demographic and mutation model parameters for the four scenarios tested using Approximate Bayesian Computation (ABC) in DiyAbc. Graphical representations of scenario topologies are presented in Fig. 5.

| Priors for the demographic parameters | |
| --- | --- |
| N1 | UN~ [10 – 750 000] |
| N2 | UN~ [10 – 750 000] |
| N3 | UN~ [10 – 750 000] |
| N_1-2_ | UN~ [10 – 750 000] |
| N_1-3_ | UN~ [10 – 750 000] |
| N_2-3_ | UN~ [10 – 750 000] |
| N_1anc_ | UN~ [10 – 750 000] |
| N_2anc_ | UN~ [10 – 750 000] |
| N_3anc_ | UN~ [10 – 750 000] |
| N_x_ | UN~ [10 – 1 500 000] |
| *t*_1_ | UN~ [10 – 350 000] |
| *t*_2_ | UN~ [10 – 350 000] |
| Constraint on parameter | *t*_2_ > *t*_1_ |
| Priors for the mutation model for microsatellites | |
| Mean mutation rate (*μ*) | LU~ [1.0 x 10^-7^ – 1.0 x 10^-3^] |
| Individual locus mutation rate | GAM ~ [1.0 x 10^-7^ – 1.0 x 10^-2^, 1.0 x 10^-4^, 2] |
| Mean coefficient (*p*) | GAM ~ [1.0 x 10^-1^ – 1.0, 0.5, 2] |
| Individual locus coefficient (*p*) | GAM ~ [1.0 x 10^-2^ – 1.0, 0.5, 2] |
| Summary statistics (SS) used in ABC analyses | |
| Mean number of alleles | |
| Mean genetic diversity | |
| Mean allele size variance | |
| Mean Garza-Williamson’s (M) for each population | |
| *F*_ST_ for each pair of populations | |

N1, effective population size of group A

N2, effective population size of group B

N3, effective population size of group C

N_1-2_, effective population size of group A under scenario III

N_1-3_, effective population size of the ancestral group A-C under scenario IV

N_2-3_, effective population size of the ancestral group B-C under scenario II

N_1anc_, effective population size of the ancestral group A under scenario II

N_2anc_, effective population size of the ancestral group B under scenario IV

N_3anc_, effective population size of the ancestral group C under scenario III

N_x_, effective population size of the most ancestral population under every scenario

*t*_1_, time (in generations=years) to the most recent divergence event under the scenarios II, III or IV

*t*_2_, time (in generations=years) to the most ancient divergence event under scenarios I, II, III or IV (see scenarios in Fig. 5)

UN, uniform distribution with two parameters [min-max]

LU, Log-uniform distribution with two parameters [min-max]

GAM, gamma distribution with four parameters [min-max, mean, shape]

**Table S4** Genetic differentiation between all populations of the scrub-legume grasshopper in southeast Iberia. Pairwise *F*_ST_ values and *F*_ST_ values corrected for null alleles are presented below and above the diagonal, respectively. Significant pairwise *F*_ST_ values after sequential Bonferroni correction are indicated in bold. Population codes are described in Table S1.

|  | RON | TEJ | PAR | PAN | MAG | ARA | OTE | RAG | GAD | BAZ | FIL | MAR | CAZ | POY | PIN | CAB | CAR | ALM | ESP |
| --- | --- | --- | --- | --- | --- | --- | --- | --- | --- | --- | --- | --- | --- | --- | --- | --- | --- | --- | --- |
| RON | - | 0.016 | 0.036 | 0.026 | 0.028 | 0.027 | 0.078 | 0.094 | 0.098 | 0.060 | 0.076 | 0.083 | 0.049 | 0.062 | 0.049 | 0.076 | 0.063 | 0.055 | 0.097 |
| TEJ | 0.026 | - | 0.040 | 0.006 | 0.015 | 0.016 | 0.060 | 0.080 | 0.079 | 0.029 | 0.054 | 0.060 | 0.032 | 0.047 | 0.035 | 0.036 | 0.048 | 0.037 | 0.076 |
| PAR | **0.046** | **0.053** | - | 0.044 | 0.064 | 0.051 | 0.122 | 0.144 | 0.144 | 0.094 | 0.117 | 0.126 | 0.084 | 0.100 | 0.093 | 0.108 | 0.099 | 0.104 | 0.136 |
| PAN | **0.041** | 0.014 | **0.054** | - | 0.012 | 0.016 | 0.052 | 0.062 | 0.067 | 0.022 | 0.040 | 0.044 | 0.025 | 0.033 | 0.027 | 0.029 | 0.037 | 0.025 | 0.063 |
| MAG | **0.039** | **0.025** | **0.078** | 0.022 | - | 0.024 | 0.055 | 0.060 | 0.055 | 0.024 | 0.037 | 0.050 | 0.027 | 0.038 | 0.031 | 0.034 | 0.037 | 0.026 | 0.071 |
| ARA | **0.045** | **0.033** | **0.069** | 0.030 | **0.040** | - | 0.055 | 0.073 | 0.065 | 0.033 | 0.054 | 0.056 | 0.036 | 0.041 | 0.040 | 0.041 | 0.046 | 0.039 | 0.079 |
| OTE | **0.096** | **0.077** | **0.141** | **0.070** | **0.066** | **0.071** | - | 0.033 | 0.025 | 0.034 | 0.033 | 0.026 | 0.034 | 0.035 | 0.033 | 0.051 | 0.043 | 0.041 | 0.049 |
| RAG | **0.119** | **0.099** | **0.164** | **0.079** | **0.079** | **0.095** | **0.044** | - | 0.022 | 0.039 | 0.030 | 0.036 | 0.040 | 0.050 | 0.038 | 0.048 | 0.048 | 0.051 | 0.055 |
| GAD | **0.113** | **0.096** | **0.159** | **0.077** | **0.069** | **0.086** | **0.041** | 0.028 | - | 0.036 | 0.015 | 0.020 | 0.027 | 0.032 | 0.025 | 0.032 | 0.026 | 0.033 | 0.035 |
| BAZ | **0.083** | **0.040** | **0.111** | **0.037** | **0.034** | **0.049** | **0.055** | **0.058** | **0.047** | - | 0.014 | 0.026 | 0.037 | 0.037 | 0.034 | 0.021 | 0.034 | 0.025 | 0.066 |
| FIL | **0.097** | **0.067** | **0.130** | **0.052** | **0.048** | **0.072** | **0.046** | **0.045** | 0.022 | 0.021 | - | 0.018 | 0.028 | 0.024 | 0.027 | 0.024 | 0.019 | 0.027 | 0.051 |
| MAR | **0.108** | **0.079** | **0.148** | **0.063** | **0.065** | **0.074** | **0.039** | **0.054** | 0.037 | **0.040** | **0.029** | - | 0.015 | 0.016 | 0.016 | 0.023 | 0.017 | 0.020 | 0.020 |
| CAZ | **0.068** | **0.050** | **0.098** | **0.043** | **0.040** | **0.057** | **0.046** | **0.049** | 0.037 | **0.052** | **0.041** | **0.031** | - | 0.015 | 0.003 | 0.023 | 0.015 | 0.014 | 0.024 |
| POY | **0.089** | **0.068** | **0.124** | **0.050** | **0.050** | **0.068** | **0.048** | **0.068** | **0.045** | **0.055** | **0.032** | 0.024 | 0.028 | - | 0.005 | 0.013 | 0.007 | 0.013 | 0.027 |
| PIN | **0.069** | **0.053** | **0.108** | **0.045** | **0.046** | **0.060** | **0.046** | **0.052** | 0.030 | **0.050** | **0.036** | **0.027** | 0.015 | 0.015 | - | 0.009 | 0.008 | 0.004 | 0.021 |
| CAB | **0.098** | **0.057** | **0.129** | 0.045 | **0.050** | **0.057** | **0.060** | **0.056** | 0.044 | 0.029 | 0.031 | 0.038 | 0.039 | 0.027 | 0.017 | - | 0.023 | 0.006 | 0.024 |
| CAR | **0.080** | **0.060** | **0.111** | **0.049** | **0.048** | **0.066** | **0.057** | **0.064** | **0.041** | **0.044** | **0.027** | **0.027** | **0.026** | 0.013 | 0.012 | 0.033 | - | 0.011 | 0.032 |
| ALM | **0.075** | **0.057** | **0.120** | **0.041** | **0.039** | **0.062** | **0.051** | **0.067** | 0.041 | **0.044** | **0.037** | **0.033** | 0.027 | 0.022 | 0.013 | 0.022 | 0.015 | - | 0.045 |
| ESP | **0.109** | **0.087** | **0.148** | **0.074** | **0.082** | **0.096** | **0.061** | **0.069** | **0.049** | **0.073** | **0.060** | **0.030** | **0.034** | **0.034** | **0.029** | 0.026 | **0.039** | **0.048** | - |

.

**Table S5** Generalized linear models (GLM) testing the association between allelic richness (*A*_R_, standardized for sample size) in populations of the scrub-legume grasshopper and current climate suitability (*HS*_CUR_), LGM climate suitability (*HS*_LGM_), and climate suitability stability (*HS*_STA_) estimated at three different spatial scales (1, 10 and 100 km^2^) around sampling localities. Longitude and latitude were also included as covariates in the models. Details about model selection are given for the best ranked equivalent models (∆AIC_c_ ≤ 2). For each model we indicate *K*, number of parameters in the model; AIC_c_, corrected Akaike’s information criterion (AIC) value; ∆AIC_c_, difference in AIC_c_ value from that of the strongest model; ω*i*, AIC_c_ weight. Model averaging was performed for the best ranked equivalent models (∆AIC_c_ ≤ 2) in order to obtain parameter estimates and unconditional standard errors (S.E.). The relative importance of each predictor is indicated (∑ω*_i_*, sum of Akaike weights of models with ∆AIC_c_ ≤ 2 in which the predictor was present).

| Model | Model  parameters | *K* | AIC_c_ | ∆AIC_c_ | ω*_i_* | Estimate  ± SE | ∑ω*_i_* | Lower  95% CI | Upper  95% CI |
| --- | --- | --- | --- | --- | --- | --- | --- | --- | --- |
| *A*_R_ (1 km^2^) | |  |  |  |  |  |  |  |  |
| 1 | Latitude | 1 | 16.90 | 0.00 | 0.418 | 0.10 ± 0.15 | 0.42 | -0.20 | 0.41 |
| 2 | Null model | 0 | 16.91 | 0.01 | 0.416 |  |  |  |  |
| 3 | *HS*_LGM_ | 1 | 18.76 | 1.86 | 0.165 | 0.05 ± 0.19 | 0.17 | -0.31 | 0.42 |
|  |  |  |  |  |  |  |  |  |  |
| *A*_R_ (10 km^2^) | |  |  |  |  |  |  |  |  |
| 1 | Latitude | 1 | 16.90 | 0.00 | 0.300 | 0.07 ± 0.42 | 0.30 | -0.20 | 0.35 |
| 2 | Null model | 0 | 16.91 | 0.01 | 0.299 |  |  |  |  |
| 3 | *HS*_STA_ | 1 | 18.47 | 1.57 | 0.137 | 0.02 ± 0.09 | 0.14 | -0.15 | 0.20 |
| 4 | *HS*_LGM_ | 1 | 18.51 | 1.61 | 0.134 | 0.04 ± 0.17 | 0.13 | -0.28 | 0.38 |
| 5 | *HS*_CUR_ | 1 | 18.58 | 1.68 | 0.130 | 0.04 ± 0.16 | 0.13 | -0.28 | 0.37 |
|  |  |  |  |  |  |  |  |  |  |
| *A*_R_ (100 km^2^) | |  |  |  |  |  |  |  |  |
| 1 | Latitude | 1 | 16.90 | 0.00 | 0.269 | 0.06 ± 0.13 | 0.27 | -0.20 | 0.33 |
| 2 | Null model | 0 | 16.91 | 0.01 | 0.268 |  |  |  |  |
| 3 | *HS*_STA_ | 1 | 17.47 | 0.57 | 0.203 | 0.03 ± 0.11 | 0.20 | -0.18 | 0.25 |
| 4 | *HS*_LGM_ | 1 | 18.03 | 1.13 | 0.153 | 0.09 ± 0.24 | 0.15 | -0.38 | 0.58 |
| 5 | *HS*_CUR_ | 1 | 18.74 | 1.84 | 0.107 | 0.04 ± 0.18 | 0.11 | -0.31 | 0.40 |

**Table S6** Generalized linear models (GLM) testing the association between the genetic admixture (*G*_ADMIX_) in populations of the scrub-legume grasshopper and climate suitability stability (*HS*_STA_) estimated at three different spatial scales (1, 10 and 100 km^2^) around sampling localities. Genetic admixture was estimated on the basis of Structure analyses for *K* = 2 (left) and *K* = 3 (right).Longitude and latitude were also included as covariates in the models. Details about model selection are given for the best ranked equivalent models (∆AIC_c_ ≤ 2). For each model we indicate *K*, number of parameters in the model; AIC_c_, corrected Akaike’s information criterion (AIC) value; ∆AIC_c_, difference in AIC_c_ value from that of the strongest model; ω*i*, AIC_c_ weight. Model averaging was performed for the best ranked equivalent models (∆AIC_c_ ≤ 2) in order to obtain parameter estimates and unconditional standard errors (S.E.). The relative importance of each predictor is indicated (∑ω*_i_*, sum of Akaike weights of models with ∆AIC_c_ ≤ 2 in which the predictor was present). GLMs built using *G*_ADMIX_ calculated for *K*= 4-5 provided similar results (data not shown).

| Model | Model  parameters | *K* | AIC_c_ | ∆AIC_c_ | ω*_i_* | Estimate  ± SE | ∑ω*_i_* | Lower  95% CI | Upper  95% CI |  | Model | Model  parameters | *K* | AIC_c_ | ∆AIC_c_ | ω*_i_* | Estimate  ± SE | ∑ω*_i_* | Lower  95% CI | Upper  95% CI |
| --- | --- | --- | --- | --- | --- | --- | --- | --- | --- | --- | --- | --- | --- | --- | --- | --- | --- | --- | --- | --- |
| *G*_ADMIX [_*_K_*_=2]_ (1 km^2^) | |  |  |  |  |  |  |  |  |  | *G*_ADMIX [_*_K_*_=3]_ (1 km^2^) | | |  |  |  |  |  |  |  |
| 1 | Null model | 0 | 8.60 | 0.00 | 0.711 |  |  |  |  |  | 1 | Null model | 0 | 3.90 | 0.00 | 0.550 |  |  |  |  |
| 2 | *HS*_STA_ | 1 | 10.41 | 1.81 | 0.289 | 0.04 ± 0.01 | 0.29 | -0.15 | 0.23 |  | 2 | Latitude | 1 | 5.52 | 1.62 | 0.245 | 0.02 ± 0.07 | 0.24 | -0.11 | 0.17 |
|  |  |  |  |  |  |  |  |  |  |  | 3 | Longitude | 1 | 5.88 | 1.98 | 0.205 | 0.01 ± 0.03 | 0.20 | -0.06 | 0.08 |
|  |  |  |  |  |  |  |  |  |  |  |  |  |  |  |  |  |  |  |  |  |
| *G*_ADMIX [_*_K_*_=2]_ (10 km^2^) | |  |  |  |  |  |  |  |  |  | *G*_ADMIX [_*_K_*_=3]_ (10 km^2^) | | |  |  |  |  |  |  |  |
| 1 | Null model | 0 | 8.60 | 0.00 | 0.596 |  |  |  |  |  | 1 | Null model | 0 | 3.90 | 0.00 | 0.550 |  |  |  |  |
| 2 | *HS*_STA_ | 1 | 9.38 | 0.78 | 0.404 | 0.07 ± 0.12 | 0.40 | -0.17 | 0.32 |  | 2 | Latitude | 1 | 5.52 | 1.62 | 0.245 | 0.03 ± 0.08 | 0.24 | -0.12 | 0.18 |
|  |  |  |  |  |  |  |  |  |  |  | 3 | Longitude | 1 | 5.88 | 1.98 | 0.205 | 0.01 ± 0.04 | 0.20 | -0.06 | 0.09 |
|  |  |  |  |  |  |  |  |  |  |  |  |  |  |  |  |  |  |  |  |  |
| *G*_ADMIX [_*_K_*_=2]_ (100 km^2^) | |  |  |  |  |  |  |  |  |  | *G*_ADMIX [_*_K_*_=3]_ (100 km^2^) | | |  |  |  |  |  |  |  |
| 1 | Null model | 0 | 8.60 | 0.00 | 0.643 |  |  |  |  |  | 1 | Null model | 0 | 3.90 | 0.00 | 0.550 |  |  |  |  |
| 2 | *HS*_STA_ | 1 | 9.78 | 1.18 | 0.357 | 0.06 ± 0.12 | 0.36 | -0.18 | 0.31 |  | 2 | Latitude | 1 | 5.52 | 1.62 | 0.245 | 0.03 ± 0.08 | 0.24 | -0.12 | 0.18 |
|  |  |  |  |  |  |  |  |  |  |  | 3 | Longitude | 1 | 5.88 | 1.98 | 0.205 | 0.01 ± 0.04 | 0.20 | -0.06 | 0.09 |

**Figure S1** (a) Results of Bayesian clustering analyses in Structure. Mean (± SD) log probability of the data [Ln Pr (*X*|*K*)] over 10 runs (left axis, open dots and error bars) for each *K*-value. The magnitude of Δ*K* as a function of *K* determines the best-supported number of clusters (*K* = 2) in Structure analyses (right axis and black dots). (b) Results of Bayesian clustering analyses in Tess. Mean DIC value over 20 runs for each *K*-value in Tess analyses. The minimum value of DIC before the first increase or stabilization indicates the best-supported number of clusters. (c) Results of genetic assignments of 354 individuals of scrub-legume grasshopper from southeast Iberia based on the Bayesian methods implemented in the program Structure and Tess for different numbers of genetic clusters (*K*). Each individual corresponds to a vertical bar, which is partitioned into *K*-coloured segments that represent the individual’s probability of belonging to the cluster with that colour. Black lines separate individuals from different populations.

.
